# Hydrothermally Synthesized ZnCr- and NiCr-Layered Double Hydroxides as Hydrogen Evolution Photocatalysts

**DOI:** 10.3390/molecules29092108

**Published:** 2024-05-02

**Authors:** Sergei A. Kurnosenko, Oleg I. Silyukov, Ivan A. Rodionov, Anna S. Baeva, Andrei A. Burov, Alina V. Kulagina, Silvestr S. Novikov, Irina A. Zvereva

**Affiliations:** Department of Chemical Thermodynamics and Kinetics, Institute of Chemistry, Saint Petersburg State University, 199034 Saint Petersburg, Russia; st040572@student.spbu.ru (S.A.K.); i.rodionov@spbu.ru (I.A.R.); st097655@student.spbu.ru (A.S.B.); st095097@student.spbu.ru (A.A.B.); st094540@student.spbu.ru (A.V.K.); st094940@student.spbu.ru (S.S.N.); irina.zvereva@spbu.ru (I.A.Z.)

**Keywords:** layered hydroxide, hydrothermal synthesis, crystallite size, photocatalysis, hydrogen, Langmuir–Hinshelwood kinetics

## Abstract

The layered double hydroxides (LDHs) of transition metals are of great interest as building blocks for the creation of composite photocatalytic materials for hydrogen production, environmental remediation and other applications. However, the synthesis of most LDHs is reported only by the conventional coprecipitation method, which makes it difficult to control the catalyst’s crystallinity. In the present study, ZnCr- and NiCr-LDHs have been successfully prepared using a facile hydrothermal approach. Varying the hydrothermal synthesis conditions allowed us to obtain target products with a controllable crystallite size in the range of 2–26 nm and a specific surface area of 45–83 m^2^∙g^−1^. The LDHs synthesized were investigated as photocatalysts of hydrogen generation from aqueous methanol. It was revealed that the photocatalytic activity of ZnCr-LDH samples grows monotonically with the increase in their average crystallite size, while that of NiCr-LDH ones reaches a maximum with intermediate-sized crystallites and then decreases due to the specific surface area reduction. The concentration dependence of the hydrogen evolution activity is generally consistent with the standard Langmuir–Hinshelwood model for heterogeneous catalysis. At a methanol content of 50 mol. %, the rate of hydrogen generation over ZnCr- and NiCr-LDHs reaches 88 and 41 μmol∙h^−1^∙g^−1^, respectively. The hydrothermally synthesized LDHs with enhanced crystallinity may be of interest for further fabrication of their nanosheets being promising components of new composite photocatalysts.

## 1. Introduction

In recent decades, heterogeneous photocatalysis has become one of the topical areas of chemical science due to its relevance in solving problems of renewable energy production, environmental remediation and fine organic synthesis [1]. The continuous development of hydrogen evolution photocatalysts, initiated by Fujishima and Honda’s discovery of water photoelectrolysis [2], involves both the search for new, more efficient materials and the applying of advanced modifications to well-known ones [3,4,5,6,7,8,9,10,11,12], such as titanium dioxide TiO_2_ [13,14,15,16,17]. Despite the significant progress made in this field [18,19,20,21,22,23,24,25,26], photocatalytic overall water splitting with a high enough efficiency still remains a challenging task for most materials available due to the thermodynamic and kinetic limitations of the reaction [27,28,29]. In view of this, great attention in recent studies has also been paid to the photocatalytic reforming of bioalcohols, carbohydrates and other plant biomass derivatives to obtain hydrogen with much higher quantum yields [30,31,32,33,34,35,36,37,38], as well as to the use of integrated approaches in the design of new photocatalytic systems [39,40,41].

A promising alternative to conventional TiO_2_-based materials is represented by layered oxide, hydroxide, nitride and other photocatalysts [42]. The features of a particular layered structure may improve spatial charge separation, involve the interlayer reaction zone in the photocatalytic cycle, and provide a researcher with a powerful toolbox of potential approaches to the further enhancement of photocatalytic performance, such as ion exchange, interlayer organic modification and exfoliation into nanosheets [43,44,45,46]. Typical representatives of such materials are layered double hydroxides (LDHs)—crystalline solids with a general formula [M^II^_1−x_M^III^_x_(OH)_2_]^x+^[(A^n−^)_x/n_∙yH_2_O]^x−^, whose structure is characterized by a regular alternation of brucite-like layers [M^II^_1−x_M^III^_x_(OH)_2_]^x+^ (M^II^ = Mg^2+^, Ca^2+^, Mn^2+^, Fe^2+^, Co^2+^, Ni^2+^, Cu^2+^, Zn^2+^; M^III^ = Al^3+^, Cr^3+^, Fe^3+^, Co^3+^, Ga^3+^, etc.) and interlayer spaces occupied by anions A^n−^ and intercalated water molecules [47]. The attractiveness of LDHs as photocatalysts is supposed to be due to their compositional flexibility, tunable band gap, ease of synthesis and relatively low cost [48,49,50,51,52,53]. In addition, many LDHs have been revealed to possess band edge potentials that are suitable for hydrogen evolution and a bandgap energy E_g_ < 3 eV, allowing them to utilize visible light [54,55,56]. Regarding the objects of this study, ZnCr-and NiCr-LDHs have already been tested in the reactions of hydrogen [57,58,59,60] and oxygen [61,62,63] generation, decomposition of organic dyes [64,65,66,67], insecticides [68] and antibiotics [69,70] as well as reduction of olefins by hydrazine [71].

However, some important aspects of LDH-based photocatalyst creation remain underdeveloped or questionable. For instance, the synthesis of most LDHs is usually conducted via the conventional coprecipitation method with sodium hydroxide as a precipitant [72,73,74,75,76,77,78,79], which makes it difficult to control the product’s crystallinity and morphology being of high significance for photocatalytic applications. In view of this, a preferable alternative is hydrothermal synthesis using urea [80,81,82] or, better yet, hexamethylenetetramine (HMT) [83], which slowly hydrolyzes upon heating, gradually increasing the pH of the reaction medium and creating favorable conditions for LDH crystal growth. Nevertheless, the HMT-assisted method has been investigated in detail only for MgAl-LDH [83]. Another more important problem is the inconsistency of the literature data on a bandgap energy of ZnCr- and NiCr-LDHs and, consequently, their intrinsic light absorption range. For instance, the studies [57,59,71] claim that the bandgap energy of ZnCr-LDHs falls in the range of 2.0–2.5 eV, which allows the corresponding photocatalysts to function under purely visible light. At the same time, the authors of another report [84] state that ZnCr-LDH has a bandgap energy of about 3.9 eV, allowing it to utilize only ultraviolet irradiation. Similar contradictions can be found between the values for NiCr-LDHs, which range from 2 [71] to 4 eV [70]. The hydrogen evolution performance of ZnCr-LDHs also differs extremely widely. For instance, the paper [85] reveals that unmodified ZnCr-LDH is not active under visible light at all, while, according to another publication [58], it exhibits an incredibly high hydrogen generation rate of about 60 mmol∙h^−1^∙g^−1^. In addition, there is little data in the literature on the dependence of LDHs’ hydrogen evolution activity on the concentration of the reaction solution. Such a dependence can usually be satisfactorily described by the conventional Langmuir–Hinshelwood model adopted from standard heterogeneous catalysis [86]. This model assumes that the reactant’s adsorption by the catalyst is mono-layer and equilibrium while the catalyst surface is homogeneous, that is, all adsorption centers on it are completely equivalent and equally accessible to the reactant molecules. That being said, the filling of the reaction centers does not affect the properties of the still vacant sites. Within these assumptions, the concentration dependence of the reaction rate resembles the classic Langmuir adsorption isotherm: its value first rises almost linearly with the increasing reactant concentration, then the slope of the graph gradually decreases and the curve reaches a plateau. This course of the dependence corresponds to the gradual filling of the surface with reactant molecules: when all the active centers are occupied, the reaction rate cannot be additionally improved by increasing the reactant content in the solution. The practical significance of obtaining such dependencies is that they make it possible to establish the optimal concentration of the reaction solution to achieve the highest rate of the target reaction (in particular, of hydrogen evolution).

With all this in mind, the present study focuses on the development and optimization of the HMT-assisted hydrothermal method for the synthesis of ZnCr- and NiCr-LDHs as hydrogen evolution photocatalysts. This study pays special attention to the correlation between synthesis conditions, the crystallinity of the LDHs and their photocatalytic performance, as well as considering the dependence of the latter on the aqueous methanol concentration within the framework of Langmuir–Hinshelwood kinetics.

## 2. Results and Discussion

### 2.1. Characterization of ZnCr- and NiCr-LDHs

In the present study, ZnCr- and NiCr-LDHs have been synthesized by a facile hydrothermal method, using HMT as an alkalizing agent. A key advantage of this approach over conventional coprecipitation with sodium hydroxide is the homogeneity in the composition of the solution during the formation of LDHs due to gradual HMT hydrolysis, accompanied by the release of ammonia, which should be more favorable for LDH crystal growth.

**Figure 1:**
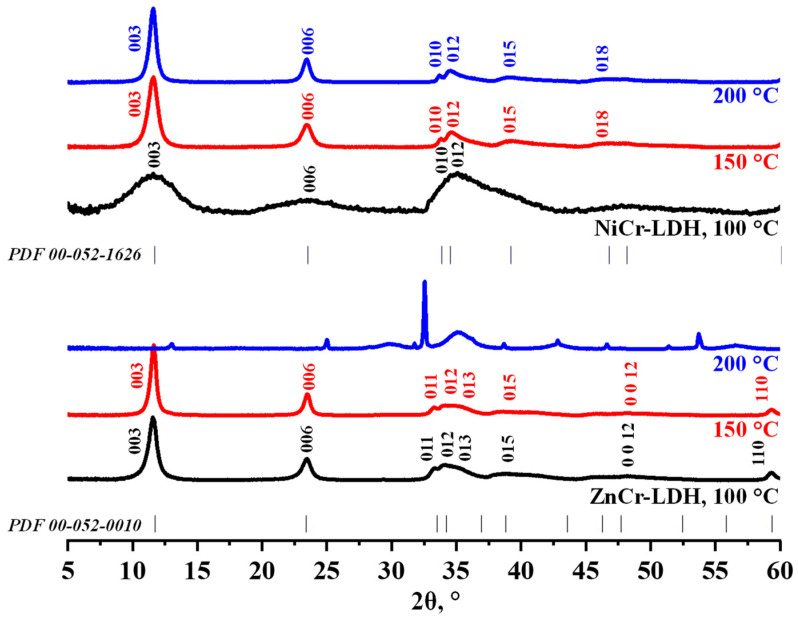
XRD patterns of ZnCr- and NiCr-LDHs synthesized at various temperature.

The ZnCr- and NiCr-LDHs were initially synthesized under the conditions generally adopted from the publication on MgAl-LDH [83], with the cation ratio M^II^:M^III^ = 2:1, implying heating of the aqueous solutions of the metal nitrates with a 3-fold HMT excess at a rate of 150 °C∙h^−1^, up to 150 °C, followed by isothermal maintenance for 1 d and cooling down for approximately 1 h (the default conditions). The resulting samples were identified by means of an XRD analysis (Figure 1, red patterns). It was shown that both LDHs are single-phase compounds not containing any crystalline impurities and that their patterns are similar to those of their counterparts synthesized by the coprecipitation method (PDF cards N° 00-052-0010 and N° 00-052-1626). The XRD patterns were successfully indexed in a hexagonal system and the lattice parameters were found to be *a* = 3.11 Å, *c* = 22.7 Å and *a* = 3.07 Å, *c* = 22.7 Å for the ZnCr- and NiCr-LDHs, respectively. The greater *a* lattice parameter of ZnCr-LDH is apparently associated with a larger Zn^2+^ crystallographic radius compared to that of Ni^2+^. At the same time, the *c* parameters of both samples are equal, indicating that their interlayer spaces have approximately the same width. With all that said, the HMT-assisted method of hydrothermal synthesis proved to be fully applicable to the LDHs under consideration.

Since the crystallinity of heterogeneous photocatalysts is one of key factors affecting their activity, subsequent experiments aimed at studying the relationship between their hydrothermal synthesis conditions (Table 1) and average crystallite size L.

**Figure 2:**
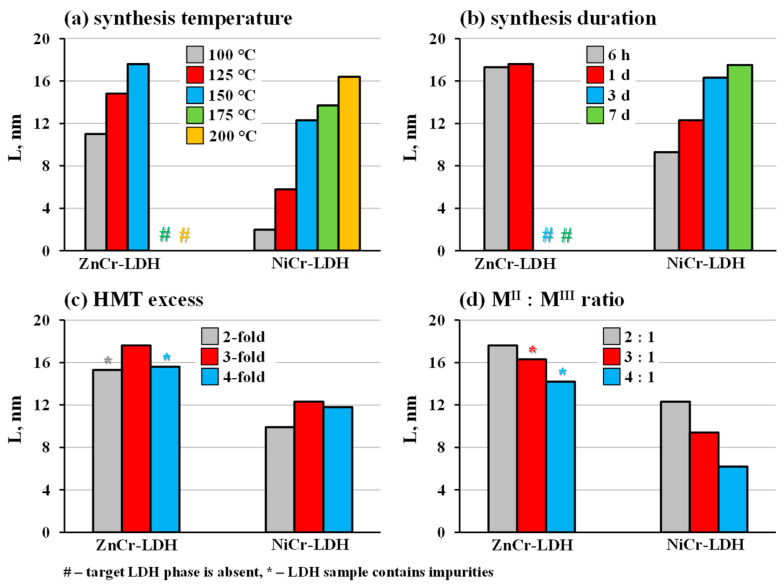
Dependence of average crystallite size on hydrothermal synthesis conditions: temperature (**a**), duration (**b**), HMT excess (**c**) and cation ratio (**d**).

The XRD patterns of all the samples obtained are presented in Figure S1 and the correlation between their synthesis conditions and resulting crystallinity is shown in Figure 2. In the first series of experiments, the synthesis temperature was chosen as the variable parameter (Figure 2a). It was shown that, for both LDHs, the average crystallite size rises monotonically with increasing temperature, from 11.0 nm at 100 °C to 17.6 nm at 150 °C for ZnCr-LDH and from 2.0 nm at 100 °C to 16.4 nm at 200 °C for NiCr-LDH, which is seen from the narrowing of the reflections at half maximum. However, the synthesis of ZnCr-LDH appears to be infeasible at temperatures above 150 °C: the XRD patterns of the products obtained at 175 °C and 200 °C do not exhibit any reflections from the target layered phase but show those of ZnCr_2_O_4_ and Cr_2_O_3_ (Figure 1 and Figure S1), indicating that the target LDH is instable in this temperature range. On the contrary, all the NiCr-LDH samples prepared at temperatures of 100–200 °C do not contain perceptible by-phases. The second series of experiments was devoted to the influence of the synthesis duration at a constant temperature of 150 °C (Figure 2b). In the case of ZnCr-LDHs, a heating elongation from 6 h to 1 d leads to a relatively small increase in crystallite size, from 17.3 to 17.6 nm, and longer experiments result in the target product decomposition into ZnCr_2_O_4_ and Cr_2_O_3_ (Figure S1). Meanwhile, the crystallite size of NiCr-LDHs increases monotonically with the synthesis duration, from 9.3 nm at 6 h to 17.5 nm at 7 d. Concerning HMT loading (Figure 2c), the 3-fold excess turned out to be optimal in terms of the crystallinity and phase purity of the products, while the use of the 2- or 4-fold excess was shown to give a smaller crystallite size and, in the case of ZnCr-LDHs, cause the formation of undesirable by-phases. An increase in the M^II^:M^III^ ratio is accompanied by the gradual crystallite size reduction from 17.6 nm at 2:1 to 14.2 nm at 4:1 for ZnCr-LDHs and from 12.3 nm at 2:1 to 6.2 nm at 4:1 for NiCr-LDHs (Figure 2d). The highest crystallinity was achieved via combining the optimal temperature (150 °C for ZnCr- and 200 °C for NiCr-LDH) with slow heating (25 °C∙h^−1^) and a continuous stirring of the reaction mixture: 18.0 nm crystallites were obtained for ZnCr-LDH and 25.7 nm ones for NiCr-LDH. Generally, the data obtained show that the HMT-assisted hydrothermal synthesis of pure single-phase ZnCr-LDH is feasible in a narrower range of conditions than that of NiCr-LDH. Nevertheless, their variation allows one to prepare the samples with different crystallite sizes.

Further in-depth investigations and photocatalytic tests were performed for five selected representatives of each LDH (M^II^:M^III^ = 2:1) with various average crystallite sizes: 11.0–18.0 for ZnCr- and 2.0–25.7 for NiCr-LDH (Table 2).

**Figure 3:**
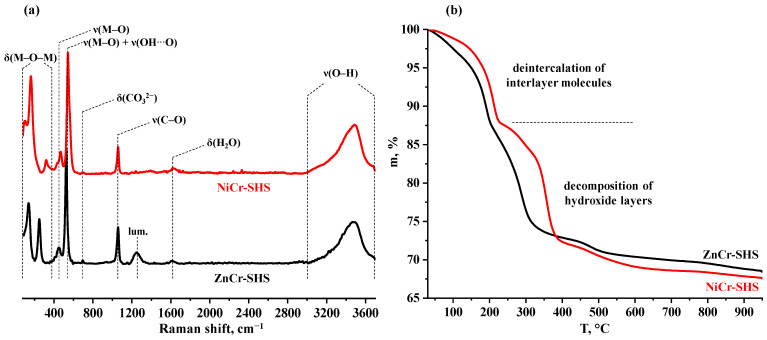
Raman spectra (**a**) and TG curves (**b**) of ZnCr- and NiCr-LDHs.

Prior to the quantitative analysis, the LDHs obtained were examined by means of Raman spectroscopy (Figure 3a). The low-frequency region of the spectra (80–600 cm^−1^) is represented by a set of bands corresponding to the metal–oxygen vibrational modes located in the brucite-like layer [87,88,89,90,91,92,93,94]. In the case of ZnCr-LDH, these bands are offset by 20–70 cm^−1^ to the low-frequency area compared to those of NiCr-LDH, which, to a zero approximation, may be explained by a greater zinc atomic mass. The Raman peaks at 695 and 1059 cm^−1^ should be attributed to the bending and stretching modes of the interlayer carbonate anions, respectively [87]. In addition, the spectra show low-intensity peaks of water bending (1620 cm^−1^) and wide asymmetric peaks of the hydroxyl group stretching (3000–3700 cm^−1^) belonging to the brucite-like layer and intercalated water molecules. At the same time, the spectra do not exhibit a 1044 cm^−1^ band that could correspond to nitrate anions [87]. Thus, the latter appear to be absent in the interlayer space or, at least, their content is significantly lower than that of the carbonates. Moreover, bands of carbon–hydrogen stretching (2800–3100 cm^−1^) are not observed, indicating that the final LDH samples do not contain perceptible amounts of residual HMT or the organic products of its hydrolysis and oxidation.

The quantitative compositions of the LDHs synthesized were determined using a combination of EDX, ICP-AES, CHN- and TG analyses (Table 2). The actual M^II^:M^III^ cation ratios measured by the first two methods were found to be in good agreement with each other and correspond to the expected value of 2:1. The elemental CHN analysis revealed an absence of nitrogen in the samples and a carbon content of 1.9–2.0%. According to the aforementioned Raman spectra (Figure 3a), the LDHs in question are free of residual organics, which allows for the attributing of all the carbon detected to the interlayer carbonate anions. In this case, their content per formula unit [M^II^_2_M^III^(OH)_6_]^+^ is 0.5 and, thereby, the interlayer space does not contain nitrates and other anions compensating for the brucite-like layer’s charge. This result is fully consistent with the data of the Raman spectroscopy (Figure 3a), pointing to the absence of nitrates in the samples. To find the amount of intercalated water (y), the LDHs were additionally studied by means of TG (Figure 3b). The curves obtained demonstrate several overlapping mass loss stages during which the samples should undergo a deintercalation of the interlayer molecules and decomposition of the brucite-like layers. The strict attribution of these stages to the liberation of water and carbon dioxide cannot be made without additional investigations. However, if the cationic and anionic compositions are known and the final thermolysis products are assumed to be Zn_2_CrO_3.5_ and Ni_2_CrO_3.5_, the difference between the total experimental and theoretical mass losses corresponds to the required water amount (y). Taking all this into account, the quantitative compositions of the LDHs prepared are [Zn_2_Cr(OH)_6_]^+^[(CO_3_)_0.5_∙yH_2_O]^−^ and [Ni_2_Cr(OH)_6_]^+^[(CO_3_)_0.5_∙yH_2_O]^−^, where y = 1.1–2.3 depending on specific synthesis conditions (Table 2).

**Figure 4:**
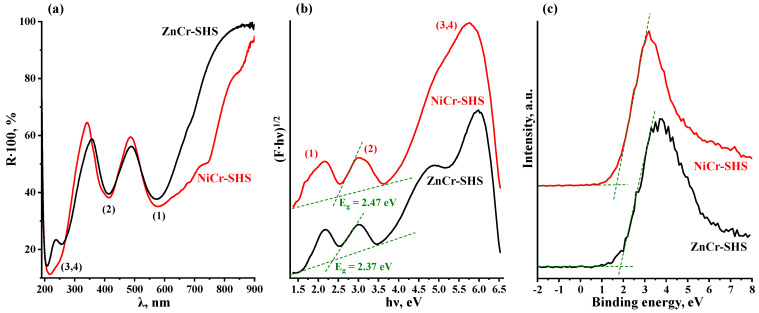
Diffuse reflectance spectra (**a**), corresponding Kubelka–Munk plots (**b**) and VB-XPS (**c**) for ZnCr- and NiCr-LDHs.

The regions of intrinsic light absorption and the optical bandgap energies E_g_ of the LDHs were examined by means of DRS with a Kubelka–Munk transformation (Figure 4a,b). As noted in the introduction, the available literature provides the reader with contradictory E_g_ values for the samples in question, which range from 2 to 4 eV. Apparently, these discrepancies originate from the complex structure of the ZnCr- and NiCr-LDHs’ spectra in comparison with those of traditional TiO_2_-based photocatalysts and, as a consequence, lead to ambiguous attribution of the absorption peaks to the interband transition. Indeed, both LDHs exhibit at least two distinctive absorption bands, marked as (1) and (2) in the visible spectrum area, as well as two overlapping ones, designated as (3,4), in the ultraviolet region (Figure 4a,b).

The rigorous determination of E_g_ from these spectra may be achieved with the use of additional information on the band structure of the samples. In particular, the results of quantum chemical calculations [95,96,97] indicate that the valence band top of the LDHs is formed by oxygen 2p-orbitals and, to a lesser extent, by chromium (ZnCr-LDH) or nickel 3d-orbitals (NiCr-LDH). The conduction band predominantly consists of 3d-orbitals of chromium (ZnCr-LDH) or those of nickel with a smaller chromium contribution (NiCr-LDH). The calculated energy gap between the valence band maximum and conduction band minimum is 2.0–2.6 eV. Therefore, it is the absorption peaks (2) that should actually relate to the genuine interband transition (Figure 4a,b). Meanwhile, according to the predicted densities of electronic states [95,96,97], the overlapping peaks (3,4) should correspond to the transitions from the valence band to higher energy 3d-orbitals of chromium and 4s-orbitals of zinc. With that said, the optical bandgap energies E_g_ of the photocatalysts prepared were found to be 2.37 eV (ZnCr-SHS) and 2.47 eV (NiCr-SHS), which corresponds to long-wave intrinsic absorption edges of 523 and 502 nm, respectively. Thus, both LDHs are expected to exhibit photocatalytic activity not only under ultraviolet but also under visible irradiation.

A necessary condition for hydrogen generation over semiconductor photocatalysts is the more negative potential of the conduction band minimum relative to the proton reduction potential (0 V vs. SHE at pH = 0). To confirm this issue for the LDHs prepared, their band edge potentials were evaluated using VB-XPS and DRS data (Figure 4). The conduction band minima were established to be approximately −0.35 V and −0.60 V vs. SHE for the ZnCr- and NiCr-LDHs, respectively (Table 3). Consequently, the reactions of hydrogen evolution from aqueous solutions are thermodynamically allowed over the LDHs in question.

**Figure 5:**
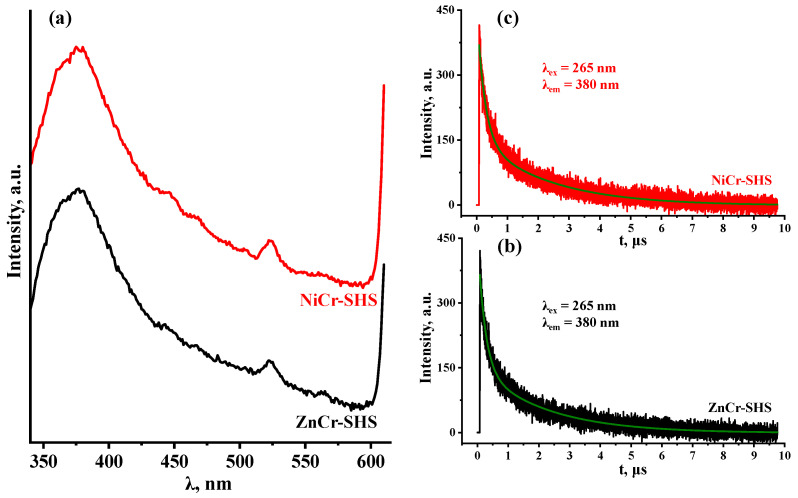
Photoluminescence spectra (**a**) and decay graphs for ZnCr- (**b**) and NiCr-LDHs (**c**).

To estimate the intensity of radiative electron–hole recombination, the most crystalline samples (ZnCr-SHS with L = 18.0 nm and NiCr-SHS with L = 25.7 nm) were investigated by means of TR-PLS (Figure 5). The same measurements were also performed for two reference photocatalysts of hydrogen production, tested earlier under the same conditions (titanium dioxide TiO_2_ P25 Degussa and layered perovskite-like oxides H_2_Ln_2_Ti_3_O_10_, Ln = La, Nd) [98] (Figure S2). All the samples were excited at λ = 265 nm and their photoluminescence decay was detected at the maximum of each emission spectrum. The average photoluminescence lifetimes of both LDHs (Table 3) were determined to be slightly less than those for TiO_2_ P25 Degussa (2.0 μs) and 2.5–3 times less than for H_2_Ln_2_Ti_3_O_10_ (4.8 μs for Ln = La, 5.4 μs for Ln = Nd). This comparison indicates that the unmodified LDHs in question are characterized by a greater electron–hole recombination rate than the aforementioned layered oxides adopting a perovskite structure.

**Figure 6:**
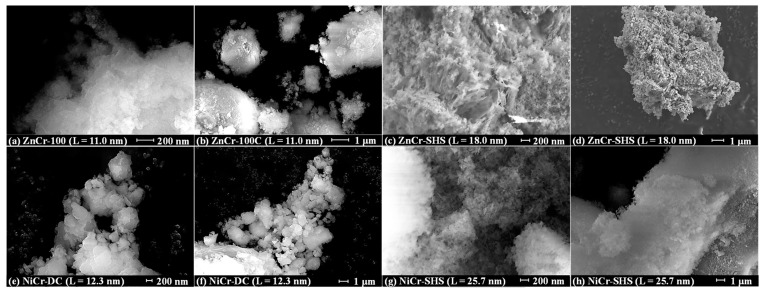
SEM images of ZnCr- (**a**–**d**) and NiCr-LDHs (**e**–**h**) with various crystallite sizes.

To study the specific surface area and particle morphology, the LDHs obtained were analyzed by means of the BET (Table 2) and SEM methods (Figure 6). It was revealed that the specific surface area of ZnCr-LDHs increases with their average crystallite size L, from 58 m^2^∙g^−1^ for ZnCr-100 (L = 11.0 nm) to 83 m^2^∙g^−1^ for ZnCr-SHS (L = 18.0 nm). Among NiCr-LDHs, on the contrary, the greatest specific surface area of 76 m^2^∙g^−1^ was observed for the sample with intermediate crystallinity, NiCr-DC (L = 12.3 nm), while the least, NiCr-100 (L = 2.0 nm), and the most crystalline samples, NiCr-SHS (L = 25.7 nm), possess a surface area of only 47 and 45 m^2^∙g^−1^, respectively. These trends cannot be explained in light of the conventional idea of a symbatic increase in crystallite and particle sizes [99,100,101] usually leading to a reduction in a specific surface area. Since the LDHs’ morphology is characterized by pronounced particle agglomeration (Figure 6), their specific surface may be rather affected by the features of these agglomerates (the number of cavities, their volume, etc.) than by the sizes of the constituent particles. For instance, the agglomerates of ZnCr-100 (L = 11.0 nm) show a relatively dense packing of the particles, while those of ZnCr-SHS (L = 18.0 nm) exhibit numerous voids, which is consistent with the greater specific surface area of the latter.

### 2.2. Photocatalytic Activity of ZnCr- and NiCr-LDHs

**Figure 7:**
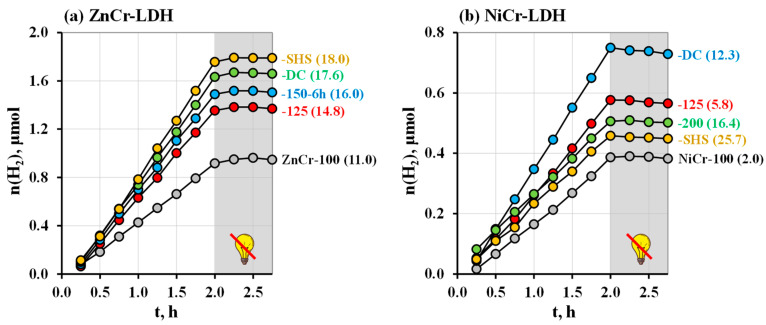
Kinetic curves of photocatalytic hydrogen evolution from 1 mol. % aqueous methanol under ultraviolet irradiation over ZnCr- (**a**) and NiCr-LDHs (**b**) with various crystallite sizes.

The hydrothermally synthesized LDHs with various average crystallite sizes have been investigated as photocatalysts of hydrogen evolution from 1 mol. % aqueous methanol under both ultraviolet and purely visible irradiation. The kinetic curves obtained under ultraviolet light (Figure 7) demonstrate linear behavior, pointing to the stable hydrogen generation rate maintained throughout the whole measurement time. When the lamp is turned off, the curves reach a plateau corresponding to a zero reaction rate.

**Figure 8:**
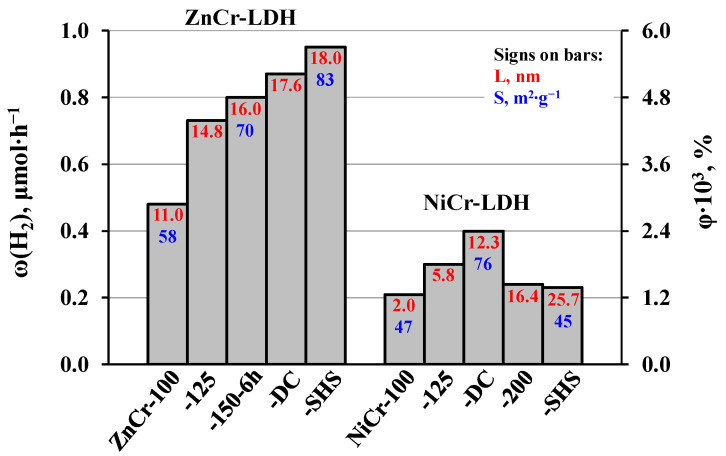
Hydrogen evolution rates and apparent quantum efficiencies achieved in 1 mol. % aqueous methanol under ultraviolet irradiation over ZnCr- and NiCr-LDHs with various crystallite sizes and specific surface areas.

In the series of ZnCr-LDHs, the hydrogen evolution activity increases monotonically with the average crystallite size, from 0.48 μmol∙h^−1^ (φ = 0.0029%) for ZnCr-100 (L = 11.0 nm) to 0.95 μmol∙h^−1^ (φ = 0.0057%) for ZnCr-SHS (L = 18.0 nm) (Figure 8). Apparently, this activity growth is caused by two key factors: the suppression of electron–hole recombination at the boundaries of the crystal lattice’s discontinuity and the enhancement of the active surface for more efficient reactant adsorption. In the series of NiCr-LDHs, the photocatalytic performance increases with crystallite size, reaches a maximum value of 0.4 μmol∙h^−1^ (φ = 0.0024%) for NiCr-DC (L = 12.3 nm), and then starts to decline (Figure 8). This trend is generally consistent with the relative change in specific surface area that appears to influence the LDH’s activity more strongly than its crystallinity. Moreover, the activity correlates with the amount of interlayer water (y) in the LDHs (Table 2), which may point to its potential involvement in the methanol oxidation, as supposed earlier for layered ion-exchangeable oxides [98,102,103,104]. On the whole, ZnCr-LDHs exhibit the 2–3 times greater activity under the chosen conditions than NiCr-LDHs.

However, both LDHs have demonstrated zero hydrogen evolution activity in aqueous methanol being irradiated by purely visible light (λ = 425 nm), despite the suitable bandgap energy. The same inactivity was reported earlier for ZnCr-LDH in a formaldehyde degradation reaction [95]. Meanwhile, several studies have reported the presence of photocatalytic activity in the visible region under conditions close to those used by us [57,58]. This fact indicates that, depending on the experimental factors (synthesis method, details of the photocatalytic measurement), these LDHs are apparently capable of generating hydrogen evolution from aqueous solutions of methyl alcohol not only under ultraviolet light. 

Although the hydrogen evolution rate over both LDHs under ultraviolet irradiation was non-zero, its value proved to be several times lower than in the case of the conventional photocatalyst TiO_2_ P25 Degussa and layered oxides H_2_Ln_2_Ti_3_O_10_ under the same conditions [98]. One of possible reasons for this may be the relatively small difference between the conduction band minimum and the proton reduction potential at neutral pH (especially in the case of ZnCr-LDH), which does not provide an enough reaction driving force. Another reason may be associated with the severe electron–hole recombination in the unmodified LDHs, which follows from the significantly lower photoluminescence lifetimes of these photocatalysts in comparison to those of other layered materials (in particular, layered perovskite-structured oxides).

**Figure 9:**
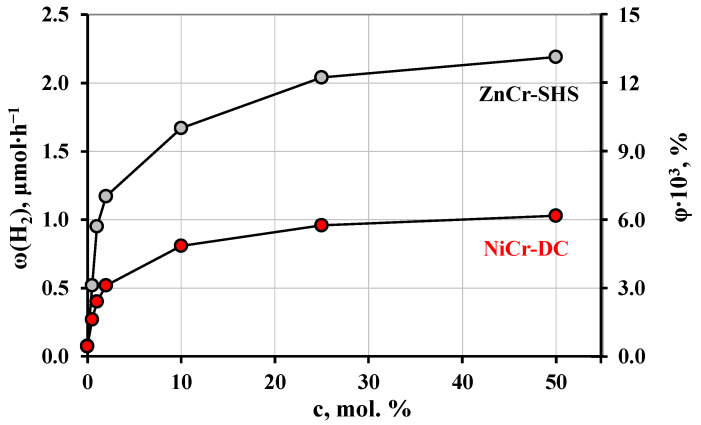
Dependence of hydrogen evolution activity of ZnCr- and NiCr-LDHs on aqueous methanol concentration.

The photocatalytic performance of two most active samples (ZnCr-SHS and NiCr-DC) was additionally evaluated in the methanol concentration range of 0–50 mol. %. Corresponding hydrogen evolution curves are shown in Figure S3. The concentration dependences obtained (Figure 9) are consistent with the standard Langmuir–Hinshelwood model for heterogeneous catalysis [86]: the hydrogen evolution rate increases sharply with the increasing methanol content in the low-concentration area and asymptotically approaches its maximum value in the high-concentration area. This behavior of the graphs is conventionally explained by the gradual filling of active centers on the catalyst surface with adsorbed reactants. In the low-concentration range, most of the active sites are vacant and the catalyst’s performance remains untapped. In the high-concentration region, on the contrary, practically all the active sites are occupied and the reaction rate cannot be boosted via the supply of excess reactants to the catalyst surface.

The highest hydrogen evolution activities achieved in 50 mol. % aqueous methanol over ZnCr- and NiCr-LDHs are 88 μmol∙h^−1^∙g^−1^ (φ = 0.013%) and 41 μmol∙h^−1^∙g^−1^ (φ = 0.0062%), respectively. Undoubtedly, these compounds demonstrate relatively low performance in comparison with the layered oxide photocatalysts tested earlier under the same conditions [98,102,103,104,105,106,107,108,109]. However, the LDHs under study differ from the aforementioned layered oxides in their intrinsic absorption of visible light, which may make them promising light-sensitive components of composite photocatalysts.

## 3. Materials and Methods

### 3.1. Hydrothermal Synthesis of LDHs with Various Crystallite sizes

The general methodology of the hydrothermal synthesis of ZnCr- and NiCr-LDHs was adopted from the study [83], where it had been developed for MgAl-LDH. The synthesis was conducted using hydrates of Zn(NO_3_)_2_ (Vekton, Saint Petersburg, Russia), Ni(NO_3_)_2_ (LenReaktiv, Saint Petersburg, Russia), Cr(NO_3_)_3_ (Chemcraft, Kaliningrad, Russia) and anhydrous hexamethylenetetramine (CH_2_)_6_N_4_ (HMT) (Vekton, Saint Petersburg, Russia). The nitrates were previously recrystallized from water and exact compositions of their hydrates were determined gravimetrically.

The selected LDH synthesis strategy is based on gradual HMT hydrolysis upon heating, with the formation of ammonia, which increases the reaction medium’s pH and results in the crystallization of the target LDHs. In the case of the cation ratio M^II^:M^III^ = 2:1, the key chemical processes occurring during the synthesis may be described by the following equations:

(CH_2_)_6_N_4_ + 10H_2_O = 6H_2_CO + 4NH_4_OH,

H_2_CO + [O] = CO_2_ + H_2_O ↔ CO_3_^2−^ + 2H^+^,

4M^II^(NO_3_)_2_ + 2M^III^(NO_3_)_3_ + 12NH_4_OH + (NH_4_)_2_CO_3_ + 2yH_2_O =

2[M^II^_2_M^III^(OH)_6_]^+^[(CO_3_)_0.5_∙yH_2_O]^−^ + 14NH_4_NO_3_,

where [O] is a generalized designation of the oxidizing agent (nitrate anion, residual air oxygen, or formaldehyde in a disproportionation reaction [110]), M^II^ = Zn or Ni and M^III^ = Cr.

The default synthesis conditions were as follows: Weights corresponding to 2.50 mmol of Zn(NO_3_)_2_ or Ni(NO_3_)_2_ and 1.25 mmol of Cr(NO_3_)_3_ hydrates (M^II^:M^III^ = 2:1) were dissolved in 10 mL of distilled water. Separately, 6.56 mmol of HMT was dissolved in 15 mL of water, which corresponds to a 3-fold HMT excess relative to the amount required for the complete alkaline hydrolysis of the nitrates. The solutions obtained were passed through membrane filters with pores of 200 nm to remove dust particles and other undesirable centers of crystallization, mixed in the 50 mL polymer vessels of steel hydrothermal reactors and purged with argon. The reactors were thoroughly shaken, heated to a temperature of 150 °C at an approximate rate of 150 °C∙h^−1^, kept for 1 d and cooled naturally for approximately 1 h to 25 °C. The solid products obtained were separated via centrifuging, rinsed with distilled water several times to achieve a supernatant pH ≈ 7 and dried in a vacuum desiccator for 1 d.

In subsequent experiments, the hydrothermal synthesis conditions were varied to investigate their influence on the crystallinity and phase purity of the final LDHs, as well as to obtain the samples with different average crystallite sizes and specific surface areas (Table 1).

### 3.2. Determination of Quantitative Composition of LDHs

The quantitative compositions of the LDHs (2:1) obtained were sought in the form [Zn_2_Cr(OH)_6_]^+^[(NO_3_)_1−x_(CO_3_)_0.5x_∙yH_2_O]^−^ and [Ni_2_Cr(OH)_6_]^+^[(NO_3_)_1−x_(CO_3_)_0.5x_∙yH_2_O]^−^, assuming that the interlayer space may only contain carbonates, nitrates and water. The cation ratio was controlled by an energy-dispersive X-ray microanalysis and inductively coupled plasma atomic emission spectroscopy. The amounts of nitrate and carbonate anions per formula unit were calculated from the nitrogen and carbon content in the samples, measured via an elemental CHN analysis. The intercalated water amount y was found from the mass loss observed during the samples’ thermolysis in air atmosphere, assuming that its final products correspond to the gross formulas Zn_2_CrO_3.5_ and Ni_2_CrO_3.5_.

### 3.3. Determination of Bandgap Energy and Band Edge Potentials

To find the bandgap energies E_g_, the diffuse reflectance spectra of the samples were transformed into coordinates (F⋅hν)^1/2^ = f(hν), where F = (1 − R)^2^∙(2R)^−1^ is the Kubelka–Munk function of a reflection coefficient R [111]. The bands of intrinsic absorption were identified using the previous theoretical investigations of the LDHs’ energy structure [95,96,97]. Low-energy linear shoulders of these bands were extrapolated to intersect the baseline and an abscissa of the intersection point was considered an optical bandgap energy E_g_.

The valance band maxima E_V_ vs. a standard hydrogen electrode (SHE) were determined using the formula E_V_ = E_V_′ + W − 4.44, where E_V_′ is an abscissa of the point obtained via the extrapolation of the linear sections of the valence band X-ray photoelectron spectrum at low binding energies, W is a work function [96] and 4.44 eV is a relative shift of electron energy scales vs. a vacuum and SHE. The conduction band minima E_C_ vs. SHE were found according to the formula E_C_ = E_V_ − E_g_ [112,113].

### 3.4. Determination of Average Photoluminescence Lifetimes

Average photoluminescence lifetimes τ were calculated based on the time-resolved photoluminescence spectra of the samples. The luminescence decay graphs were fitted by a biexponential function, I(t) = A_1_∙exp(−t/t_1_) + A_2_∙exp(−t/t_2_) + I_0_, using OriginPro 9.5 software (OriginLab Corporation, Northampton, MA, USA). The τ values were found via the formula τ = (A_1_∙t_1_^2^ + A_2_∙t_2_^2^)∙(A_1_∙t_1_ + A_2_∙t_2_)^−1^, where A_n_ and t_n_ are the amplitudes and time constants, respectively, determined during fitting [114].

### 3.5. Investigation of Photocatalytic Activity

The photocatalytic activity in the reaction of hydrogen evolution from 1 mol. % aqueous methanol was studied for 5 samples of each LDH with a different crystallite size (Table 2). The experiments were carried out under both ultraviolet irradiation (DRT-125 mercury lamp, 125 W, λ > 220 nm) and purely visible light (LED source, 100 W, λ = 425 nm) in the photocatalytic laboratory setting used in our previous reports [98,102,103,104,105,106,107,108,109] and described in detail in Information S1. The spectra of the light sources are shown in Figure S4. The photocatalytic performance of the samples was evaluated in terms of the hydrogen evolution rate ω and apparent quantum efficiency φ.

In a typical photocatalytic experiment, 25 mg of the sample was placed in a round-bottom flask containing 50 mL of 1 mol. % aqueous methanol with a pH ≈ 6. The flask was sealed, shaken and sonicated in an Elmasonic S10H bath (Elma, Singen, Germany) for 10 min. The suspension obtained was pumped into the reaction compartment of the cell, after which the magnetic stirrer, light filter and light source were turned on. The reaction suspension was purged with argon for 30 min to remove air residues and, after this, kept under irradiation for 2 h, during which the hydrogen content in the gas circuit was analyzed chromatographically at 15 min intervals. After this, the light source was turned off to organize a dark stage and make sure that the photocatalytic reaction stopped. In the end, the areas of hydrogen’s chromatographic peaks were converted to hydrogen amounts that, in turn, were used to plot kinetic curves. The latter were approximated by linear functions and differentiated to find the hydrogen evolution rates ω. The apparent quantum efficiencies of the reactions were calculated using the equation φ = 2∙ω∙f^−1^∙100%, where f is the lamp photon flux in the photocatalyst absorption range measured previously, as described in Information S2.

To investigate the dependence of the hydrogen evolution activity on the aqueous methanol concentration, a series of similar photocatalytic measurements were conducted using the LDHs that demonstrated the highest performance in the aforementioned experiments and taking 0, 0.5, 1, 2, 10, 25 and 50 mol. % methanol solutions.

### 3.6. Instrumentation

#### 3.6.1. XRD

Powder X-ray diffraction (XRD) patterns were obtained on a Rigaku Miniflex II benchtop Röntgen diffractometer (Tokyo, Japan) using Cu K_α_ radiation, an angle range 2θ = 3–60° and a scanning rate of 10°∙min^−1^. The phase composition was determined using Rigaku PDXL 2 software and the powder diffraction files (PDF) of The International Centre for Diffraction Data (ICDD). The indexing of the diffraction patterns, calculation of the lattice parameters and average crystallite sizes L were performed on the basis of all the reflections observed, using DiffracPlus Topas software (Bruker, Karlsruhe, Germany).

#### 3.6.2. Raman Spectroscopy

Raman spectra were measured on a Bruker Senterra spectrometer (Billerica, MA, USA) in the spectral range of 80–3700 cm^−1^, using a 785 nm laser (power 50 mW, single accumulation time 120 s, 8 repetitions) for ZnCr-LDHs and a 532 nm laser (power 5 mW, single accumulation time 60 s, 4 repetitions) for NiCr-LDHs. In the first case, the signal was collected in a luminescence subtraction mode and the region of 1500–3700 cm^−1^ was upscaled 10 times.

#### 3.6.3. SEM-EDX

The morphology and elemental composition of the samples were studied on a Zeiss Merlin scanning electron microscope (SEM) (Oberkochen, Germany) equipped with an Oxford Instruments INCAx-act energy-dispersive X-ray spectroscopic microanalyzer (EDX) (Abingdon, UK).

#### 3.6.4. ICP-AES

The cation ratio in the samples was additionally controlled by inductively coupled plasma atomic emission spectroscopy (ICP-AES) on a Shimadzu ICPE-9000 spectrometer (Kyoto, Japan). The samples were preliminarily dissolved in 12 M hydrochloric acid and the solutions obtained were diluted 100 times prior to their analysis.

#### 3.6.5. CHN Analysis

The nitrogen and carbon content in the samples was determined via an elemental CHN analysis on a Euro EA3028-HT analyzer (EuroVector, Pavia, Italy).

#### 3.6.6. TG

A thermogravimetric (TG) analysis was performed on a Netzsch TG 209 F1 Libra thermobalance (Selb, Germany) in a synthetic air atmosphere. The temperature program included heating from room temperature to 950 °C at a rate of 10 °C∙min^−1^, followed by a 20 min isotherm at 950 °C to achieve the establishing of a constant mass.

#### 3.6.7. DRS

Diffuse reflectance spectra (DRS) were recorded on a Persee T8DCS spectrophotometer equipped with an SI19-1 integrating sphere (Auburn, CA, USA) in the range of 190–900 nm after sample deposition on a barium sulfate substrate.

#### 3.6.8. VB-XPS

The X-ray photoelectron spectra of the valence band region (VB-XPS) were measured on a Thermo Fisher Scientific Escalab 250Xi spectrometer (Waltham, MA, USA) with a monochromatized Al K_α_ X-ray source and binding energy scale calibration on a C 1s peak at 284.8 eV.

#### 3.6.9. TR-PLS

Time-resolved photoluminescence spectroscopy (TR-PLS) was performed using a Horiba Jobin Yvon Fluorolog-3 spectrofluorometer (Kyoto, Japan). The samples were excited by a 265 nm light-emitting diode and the photoluminescence decay was measured at 380 nm.

#### 3.6.10. BET

The specific surface area S was measured on a Quadrasorb SI adsorption analyzer (Boynton Beach, FL, USA). Prior to the analysis, 100–150 mg of each sample was degassed at 25 °C for 12 h. Adsorption isotherms were measured at a liquid nitrogen temperature (−196 °C), with nitrogen as an adsorbate. The values of the specific surface areas were calculated via the conventional multipoint Brunauer–Emmett–Teller method (BET).

#### 3.6.11. pH-Metry

The pH values of the wash water during the sample rinsing after hydrothermal synthesis were monitored using a laboratory pH-meter Mettler Toledo SevenCompact S220 equipped with an InLab Expert Pro-ISM electrode (Greifensee, Switzerland).

## 4. Conclusions

The present study has demonstrated the applicability of a facile hydrothermal method to the preparation of ZnCr- and NiCr-LDHs using metal nitrates and hexamethylenetetramine as an alkalizing agent. All the syntheses resulted in either the formation of a carbonate rather than a nitrate form of the LDHs, or the formation of its decomposition products (at high temperatures). The analysis of the qualitative and quantitative compositions of the obtained samples showed that, in all cases, the carbonate was the only interlayer anion. Additionally, the resulting LDHs contained water, the amount of which depended on their synthesis conditions. Varying the synthesis conditions allowed us to obtain target samples with various average crystallite sizes of 2–26 nm and specific surface areas of 45–83 m^2^∙g^−1^. The optical bandgap energies of the LDHs were revealed to be 2.37 and 2.47 eV, which potentially allows them to utilize visible light during photocatalytic processes. However, both LDHs demonstrated non-zero photocatalytic activity in the reaction of hydrogen evolution from aqueous methanol only under ultraviolet irradiation. The performance of ZnCr-LDHs grows monotonically with their average crystallite size, while that of NiCr-LDHs reaches its maximum with intermediate-sized crystallites and then decreases due to the specific surface area reduction. The concentration dependences of the hydrogen evolution activity of both LDHs are generally consistent with the conventional Langmuir–Hinshelwood model for heterogeneous catalysis. In 50 mol. % aqueous methanol, the hydrogen generation rate over ZnCr- and NiCr-LDHs reaches 88 and 41 μmol∙h^−1^∙g^−1^, respectively. The low activity of the tested samples may be due, among other things, to the relatively high rate of the recombination of electrons and holes indicated by the photoluminescence measurements. Another possible reason for the low activity is the insufficient accessibility of the interlayer space of the carbonate form of LDHs, which is a potential reaction zone for water and methanol molecules, which causes the reaction proceeding only on the external surface of the particles. Obtaining forms of LDHs intercalated with organic molecules or exfoliated into separate nanosheets might serve as an effective way to solve this problem. Although the activities achieved for now are not very impressive compared to those of the layered oxide photocatalysts tested earlier, these hydrothermally synthesized LDHs with enhanced crystallinity may be of great interest for the further fabrication of their nanosheets and designing new composite photocatalysts, including those active under visible light.

## Figures and Tables

**Table 1:** Conditions for hydrothermal synthesis of ZnCr- and NiCr-LDHs.

Experiment Series	Temperature, °C	Duration, d	HMT Excess	M^II^:M^III^ Ratio	Heating Rate, °C∙h^−1^	Stirring Rate, rpm
Default conditions	150	1	3-fold	2:1	150	−
Variable temperature	100	1	3-fold	2:1	150	−
	125					
	150					
	175					
	200					
Variable duration	150	0.25	3-fold	2:1	150	−
		1				
		3				
		7				
Variable HMT excess	150	1	2-fold	2:1	150	−
			3-fold			
			4-fold			
Variable M^II^:M^III^ ratio	150	1	3-fold	2:1	150	−
				3:1		
				4:1		
Slow heating and stirring	150 (ZnCr)	1	3-fold	2:1	25	1000
	200 (NiCr)					

**Table 2:** Average crystallite sizes L, quantitative compositions (cation ratios M^II^:M^III^ and water amounts per formula unit y) and specific surface areas S of ZnCr- and NiCr-LDHs chosen for photocatalytic experiments.

Sample Abbreviation	L, nm	Synthesis Conditions	M^II^:M^III^ Ratio		y	S, m^2^∙g^−1^
			EDX	ICP-AES		
ZnCr-LDH						
ZnCr-100	11.0	100 °C, 1 d	2:1	2:1.01	1.1	58
ZnCr-125	14.8	125 °C, 1 d	−	−	−	−
ZnCr-150-6h	16.0	150 °C, 6 h	2:1	2:1.03	1.5	70
ZnCr-DC	17.6	150 °C, 1 d	−	−	−	−
ZnCr-SHS	18.0	150 °C, 1 d, slow heating, stirring	2:1	2:1.05	2.0	83
NiCr-LDH						
NiCr-100	2.0	100 °C, 1 d	2:1	2:0.97	2.1	47
NiCr-125	5.8	125 °C, 1 d	−	−	−	−
NiCr-DC	12.3	150 °C, 1 d	2:1	2:0.98	2.3	76
NiCr-200	16.4	200 °C, 1 d	−	−	−	−
NiCr-SHS	25.7	200 °C, 1 d, slow heating, stirring	2:1	2:0.96	1.9	45

**Table 3:** Light absorption regions, band edge potentials vs. SHE and average photoluminescence lifetimes for ZnCr- and NiCr-LDHs.

Sample	E_g_, eV	λ_max_, nm	E_V_, V	E_C_, V	τ, μs
ZnCr-SHS	2.37	523	2.02	−0.35	1.8
NiCr-SHS	2.47	502	1.87	−0.60	1.9

## Data Availability

The research data are available in the body of the article and the Supplementary Materials.

## Supplementary Materials

The following supporting information can be downloaded at https://www.mdpi.com/article/10.3390/molecules29092108/s1, Figure S1: XRD patterns of ZnCr- and NiCr-LDHs synthesized under various conditions; Figure S2: Photoluminescence spectra and decay graphs with average photoluminescence lifetimes for TiO_2_ P25 Degussa and layered perovskite-like oxides H_2_La_2_Ti_3_O_10_ and H_2_Nd_2_Ti_3_O_10_; Figure S3: Kinetic curves of photocatalytic hydrogen evolution from aqueous methanol of various concentrations over ZnCr- and NiCr-LDHs; Figure S4: Emission spectra of light sources used in photocatalytic experiments; Information S1: Scheme and operating principle of the photocatalytic setting; Information S2: Determination of the lamp photon flux.
