# Supplementary material for: Hydrothermally Synthesized ZnCr- and NiCr-Layered Double Hydroxides as Hydrogen Evolution Photocatalysts
Source: Molecules. 2024 May 2;29(9):2108. doi: 10.3390/molecules29092108 (PMC11085494; doi:10.3390/molecules29092108)
Supplement: Supplementary file 1 [file molecules-29-02108-s001.zip › molecules-2911133-supplementary.pdf]

# Supplementary materials

*Article*

# Hydrothermally synthesized ZnCr- and NiCr-layered double hydroxides as hydrogen evolution photocatalysts

**Sergei A. Kurnosenko, Oleg I. Silyukov *, Ivan A. Rodionov, Anna S. Bayova, Andrei A. Burov, Alina V. Kulagina, Silvestr S. Novikov and Irina A. Zvereva**

Department of Chemical Thermodynamics and Kinetics, Institute of Chemistry, Saint Petersburg State University, 199034 Saint Petersburg, Russia; st040572@student.spbu.ru (S.A.K.); i.rodionov@spbu.ru (I.A.R.); st097655@student.spbu.ru (A.S.B.); st095097@student.spbu.ru (A.A.B.); st094540@student.spbu.ru (A.V.K.); st094940@student.spbu.ru (S.S.N.); irina.zvereva@spbu.ru (I.A.Z.)

* Correspondence: oleg.silyukov@spbu.ru

**Figure S1**

XRD patterns of ZnCr- (a) and NiCr-LDHs (b) synthesized under variable conditions

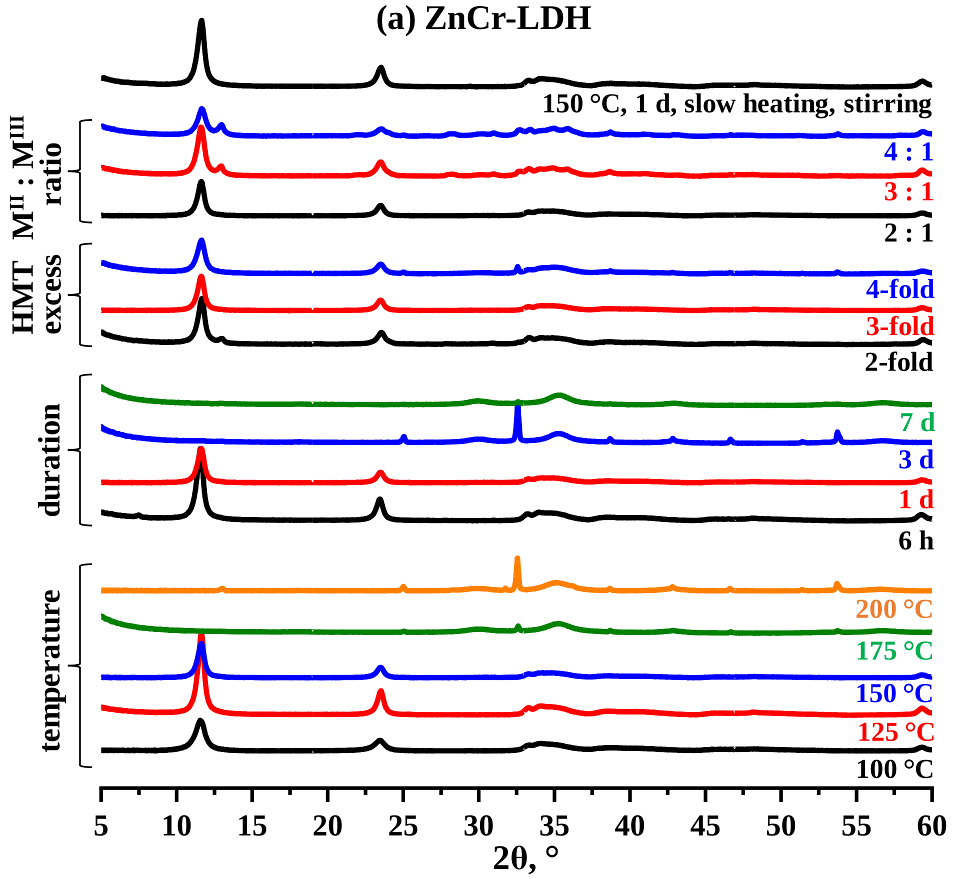


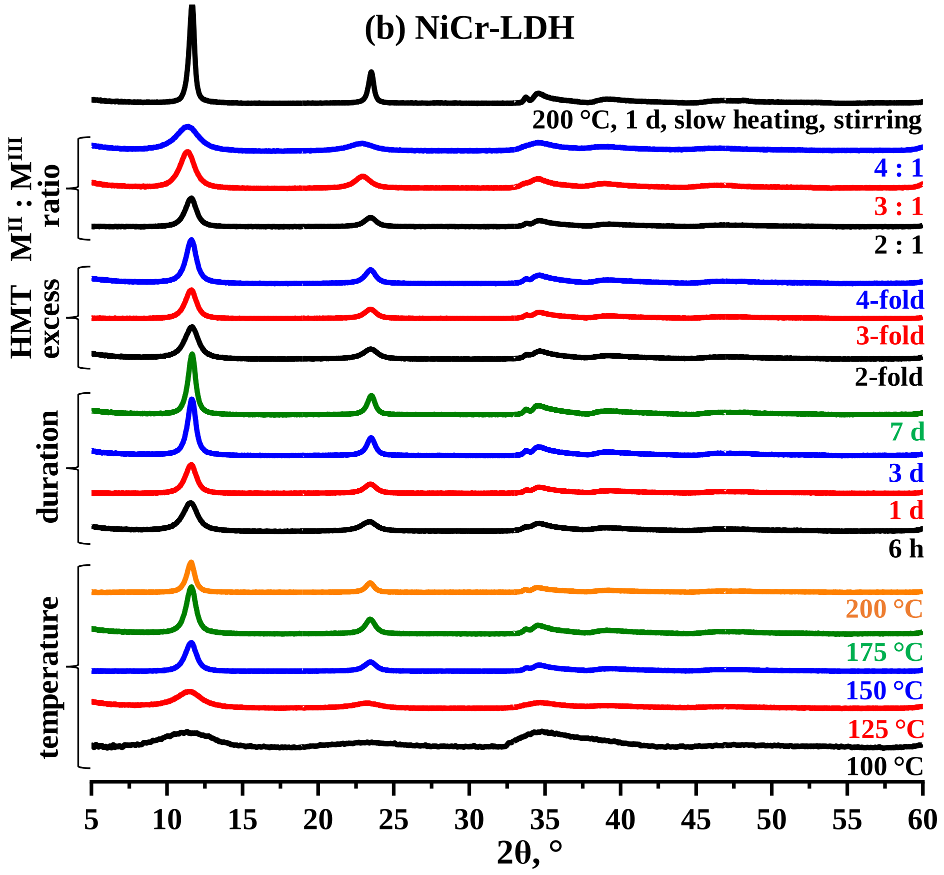

**Figure S2**

Photoluminescence spectra (a) and decay graphs with average photoluminescence lifetimes for $TiO_2$ P25 Degussa (b) and layered perovskite-like oxides $H_2La_2Ti_3O_{10}$ (c), $H_2Nd_2Ti_3O_{10}$ (d)

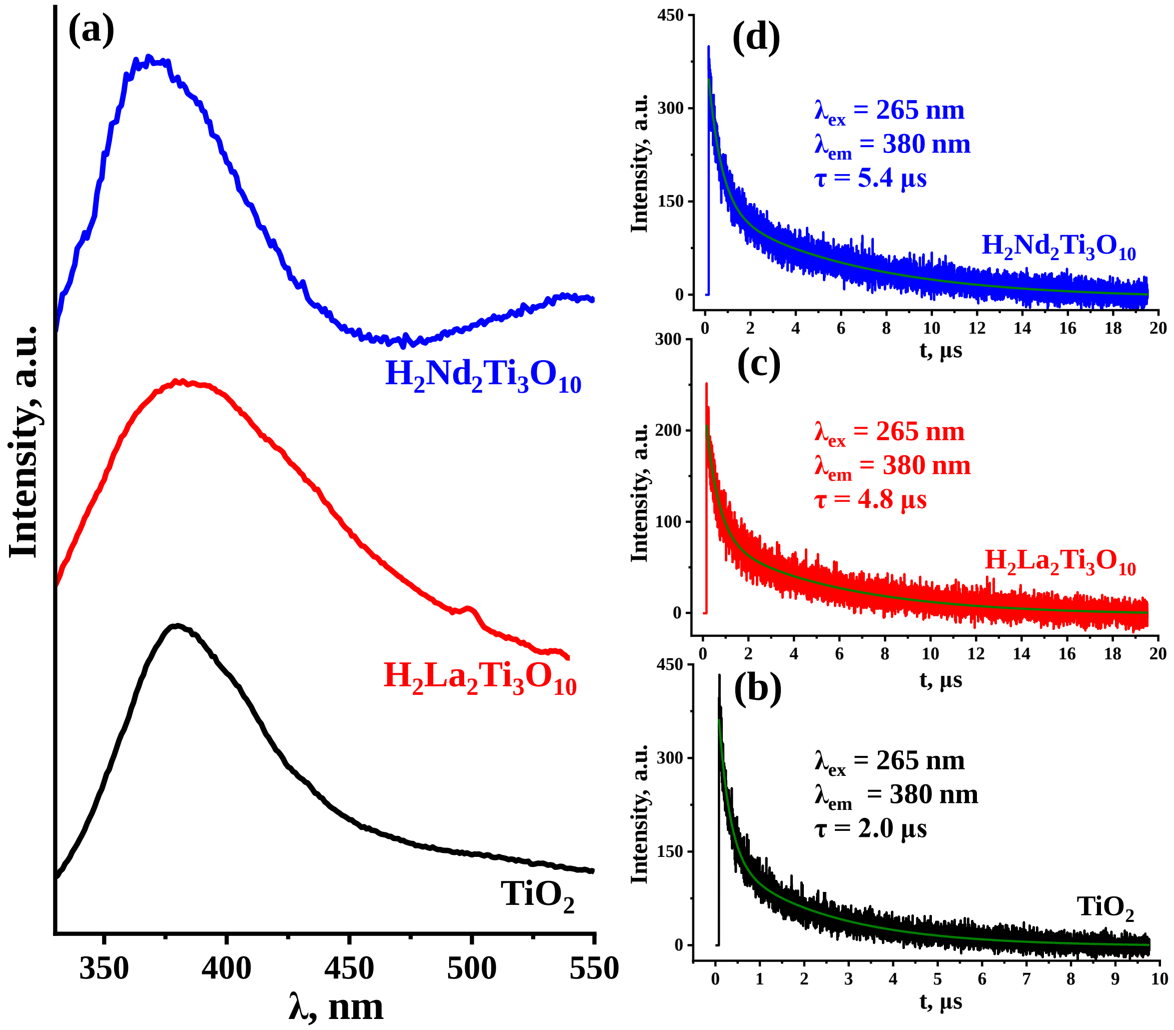

## Figure S3

Kinetic curves of photocatalytic hydrogen evolution from aqueous methanol of various concentration under ultraviolet irradiation over ZnCr- (a) and NiCr-LDHs (b)

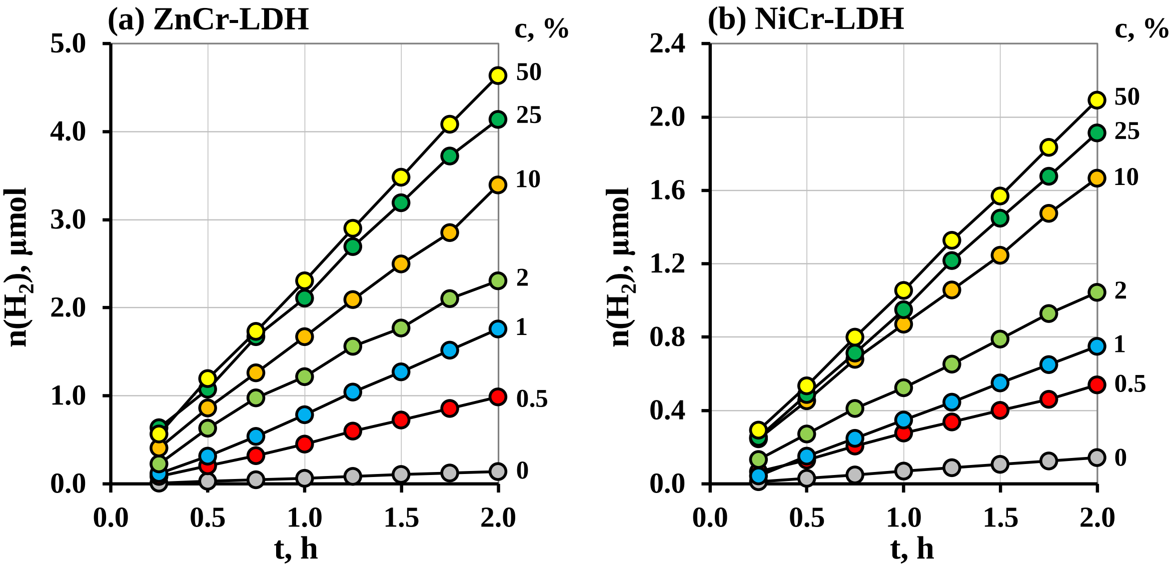

**Figure S4**

Emission spectra of light sources used in photocatalytic experiments

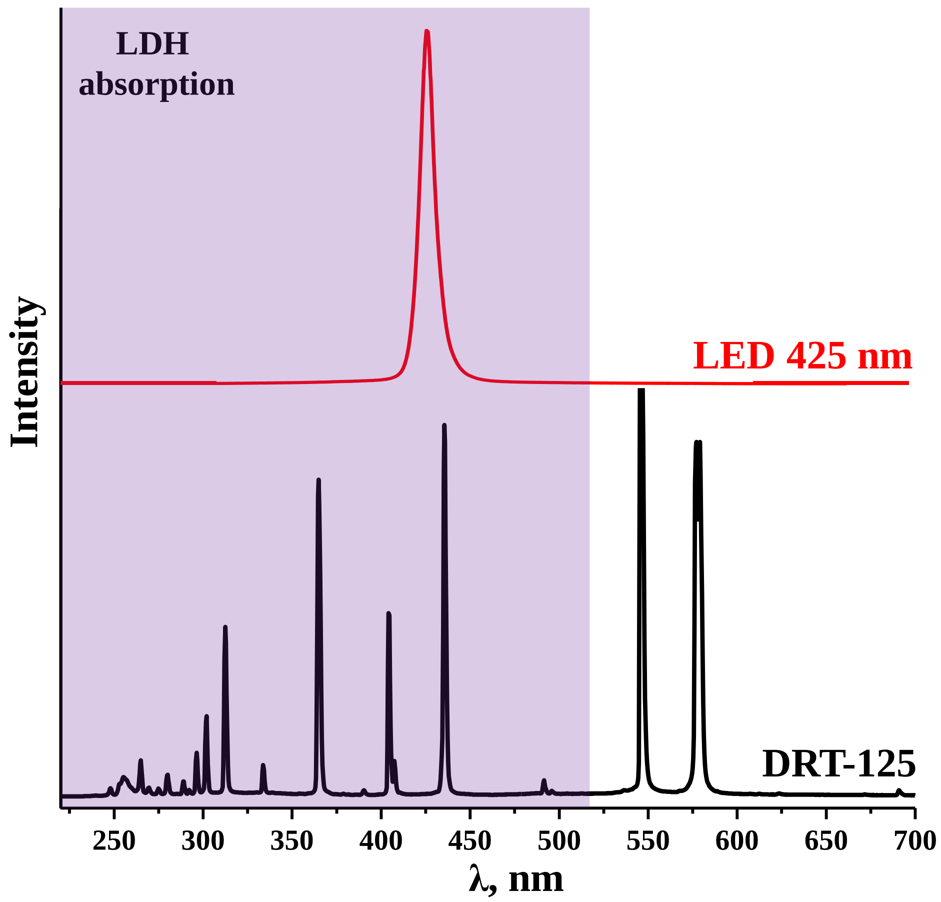

## Information S1

Scheme and operating principle of the photocatalytic setting

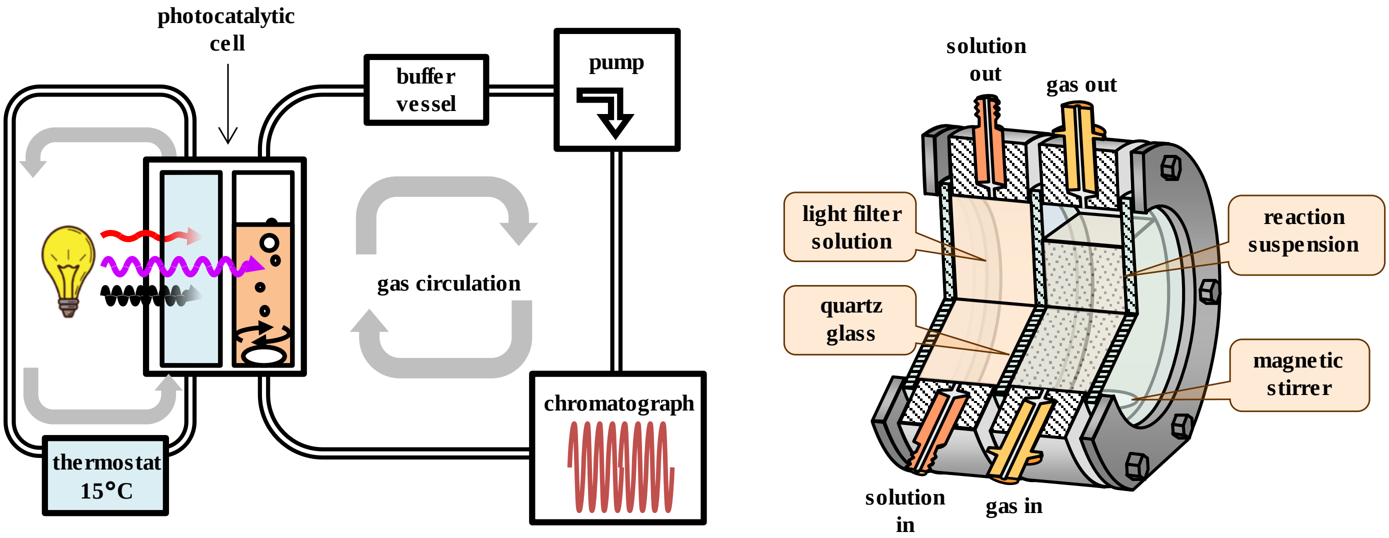


(a) (b)

Photocatalytic measurements were performed on the laboratory photocatalytic setting (a), consisting of a replaceable illuminator, reaction cell (b), online gas chromatograph and closed gas circulation system, connecting the last two components. The experiments were carried out using two types of illuminators: an arc mercury tube lamp DRT-125 (125 W), giving ultraviolet irradiation with a photon flux of 5.1 mmol·h$^{-1}$ in the photocatalyst absorption range, and a LED source (100 W) providing purely visible light with $\lambda = 425$ nm (emission spectra are shown in Figure S4). The self-made photocatalytic cell represents an external irradiation reactor, including two compartments equipped with branch pipes for the supply and removal of solutions and gases. The compartment closest to the lamp is filled with a light filtering solution of NaBr and KCl (6 g·L$^{-1}$ of each salt) circulating through the thermostat, maintaining a temperature of 15 °C. The light filter cuts of hard ultraviolet irradiation of the DRT-125 lamp ($\lambda < 220$ nm) and provides cooling of the cell. The compartment farthest from the lamp is for the reaction suspension being intensively stirred during the experiment. In the course of the photocatalytic reaction, gaseous products come from the cell into the gas circulation system. Their detection is conducted according to a specified program at fixed time intervals by a Shimadzu GC-2014 gas chromatograph (Kyoto, Japan), equipped with a 30 m long Rt-Msieve 5A capillary column, thermal conductivity detector and using argon as a carrier gas.

## Information S2

Determination of the lamp photon flux

The photon flux of the DRT-125 lamp was first measured in the range of 220–400 nm in accordance with the conventional ferrioxalate actinometry technique and then was recalculated to the range of 220–530 nm via multiplying by the ratio of integral light intensities in these regions found from the lamp spectrum (Figure S4).

Briefly, 3 g of potassium ferrioxalate $K_3[Fe(C_2O_4)_3]\cdot 3H_2O$ was dissolved in 1000 mL of 0.05 M sulfuric acid. 50 mL of the photosensitive solution prepared was pumped into the reaction compartment of the photocatalytic cell and irradiated by the DRT-125 lamp through the NaBr + KCl light filter (transmission range $\lambda > 220$ nm) for 20 s. Then 0.5 mL of the irradiated solution was sampled from the cell, mixed with 2 mL of an acetate buffer and 1 mL of 0.1% 1,10-phenanthroline and made up to the 25 mL mark with distilled water in a volumetric flask. Amount of $Fe^{2+}$ in the final solution was determined spectrophotometrically using a previously built calibration plot. The similar experiment was conducted using a $NaNO_2$ light filter (transmission range $\lambda > 400$ nm) and irradiation time 120 s. Each series of measurements was repeated 3 times and $Fe^{2+}$ contents were averaged. Assuming the quantum yield of the $Fe^{2+}$ formation from potassium ferrioxalate is 100%, we calculated the photon flux f in the active range (220–530 nm) in accordance with the formula:

$$f = V \cdot \left(\frac{c}{t} - \frac{c'}{t'}\right) \cdot \frac{\int_{220}^{530} I(\lambda)d\lambda}{\int_{220}^{400} I(\lambda)d\lambda},$$

where V is a volume of the photosensitive solution in the cell (L), c is concentration of $Fe^{2+}$ (mM) in the photosensitive solution after its irradiation through the NaBr + KCl light filter for the time t (h), c' is concentration of $Fe^{2+}$ (mM) in the photosensitive solution after its irradiation through the $NaNO_2$ light filter for the time t' (h) and I is light intensity in the lamp spectrum (Figure S4).
